# Effects of levosimendan on renal blood flow and glomerular filtration in patients with acute kidney injury after cardiac surgery: a double blind, randomized placebo-controlled study

**DOI:** 10.1186/s13054-021-03628-z

**Published:** 2021-06-12

**Authors:** Maria Tholén, Sven-Erik Ricksten, Lukas Lannemyr

**Affiliations:** Department of Anesthesiology and Intensive Care Medicine At the Sahlgrenska Academy, University of Gothenburg and Section for Cardiothoracic Anesthesia and Intensive Care, Sahlgrenska University Hospital, Blå Stråket 7, 5th Floor, 413 45 Gothenburg, Sweden

## Abstract

**Background:**

Acute kidney injury (AKI) is a common and serious complication after cardiac surgery, and current strategies aimed at treating AKI have proven ineffective. Levosimendan, an inodilatating agent, has been shown to increase renal blood flow and glomerular filtration rate in uncomplicated postoperative patients and in patients with the cardiorenal syndrome. We hypothesized that levosimendan through its specific effects on renal vasculature, a preferential vasodilating effect on preglomerular resistance vessels, could improve renal function in AKI-patients with who did not have clinical indication for inotropic support.

**Methods:**

In this single-center, double-blind, randomized controlled study, adult patients with postoperative AKI within 2 days after cardiac surgery, who were hemodynamically stable with a central venous oxygen saturation (ScvO_2_) ≥ 60% without inotropic support were eligible for inclusion. After randomization, study drug infusions, levosimendan (n = 16) or placebo (n = 13) were given for 5 h. A bolus infusion of levosimendan (12 µg/kg), were given for 30 min followed by 0.1 µg/kg/min for 5 h. Renal blood flow and glomerular filtration rate were measured using infusion clearance of para-aminohippuric acid and a filtration marker, respectively. As a safety issue, norepinephrine was administered to maintain mean arterial pressure between 70–80 mmHg. Intra-group differences were tested by Mann–Whitney U-tests, and a linear mixed model was used to test time and group interaction.

**Results:**

Twenty-nine patients completed the study. At inclusion, the mean serum creatinine was higher in the patients randomized to levosimendan (148 ± 29 vs 127 ± 22 µmol/L, *p* = 0.030), and the estimated GFR was lower (46 ± 12 vs 57 ± 11 ml/min/1.73 m^2^, *p* = 0.025). Levosimendan induced a significantly (*p* = 0.011) more pronounced increase in renal blood flow (15%) compared placebo (3%) and a more pronounced decrease in renal vascular resistance (− 18% vs. − 4%, respectively, *p* = 0.043). There was a trend for a minor increase in glomerular filtration rate with levosimendan (4.5%, *p* = 0.079), which did differ significantly from the placebo group (*p* = 0.440). The mean norepinephrine dose was increased by 82% in the levosimedan group and decreased by 29% in the placebo group (*p* = 0.012).

**Conclusions:**

In hemodynamically stable patients with AKI after cardiac surgery, levosimendan increases renal blood flow through renal vasodilatation.

Trial registration

NCT02531724, prospectly registered on 08/20/2015. https://clinicaltrials.gov/ct2/show/NCT02531724?cond=AKI&cntry=SE&age=1&draw=2&rank=1

## Introduction

Acute kidney injury (AKI) is a common complication after cardiac surgery and associated with increased morbidity and mortality [1]. Even mild AKI, noticeable only as a slight increase in serum creatinine, is associated with poor outcome [2]. The mechanisms of cardiac surgery-related AKI are likely multi-factorial, including perturbations of blood flow and oxygen delivery, hemolysis, inflammation and micro-embolization. Impaired renal oxygenation may arise from low oxygen delivery during surgery (due to perioperative hypotension, hemodilution and renal vasoconstriction) as well as postoperative low cardiac output [3, 4].

In AKI, the deterioration of renal excretory function, i.e., a reduction of the glomerular filtration rate (GFR), leads to retention of nitrogen metabolism waste products, detectable as an increased serum creatinine concentration. Thus, a reasonable goal in the management of AKI would be to improve the GFR. As there is a close relationship between GFR, tubular sodium reabsorption and renal oxygen consumption [5–7], a drug-induced increase in GFR will, however, increase renal oxygen consumption which could potentially impair renal oxygenation (i.e. the renal oxygen demand/supply relationship) further. Renal oxygenation is severely impaired in patients with early cardiac surgery-associated AKI [8], in turn, caused by a substantial increase (50%) in renal vascular resistance and a 40% lower renal blood flow (RBF), compared with post-cardiac surgery patients with no AKI. In this situation, an ideal agent to treat AKI, would be one that induces a vasodilation of preferentially the preglomerular resistance vessels increasing both RBF and GFR. Such an agent will not only increase GFR but would also meet the increased renal metabolic demand by increased renal oxygen delivery.

Levosimendan is a calcium sensitizing agent used as an inodilator in cardiac failure. Although three major trials have failed to demonstrate a superiority of levosimendan over placebo regarding prevention of major adverse outcomes after cardiac surgery such as acute kidney injury [9–11], other studies have shown that levosimendan may have beneficial effects on renal function in several clinical situations [12–15]. More specifically, levosimendan increases RBF through renal vasodilation with preference for the afferent arterioles, resulting in higher intra-glomerular pressure and increased filtration [16, 17]. Thus, through direct vascular effects, levosimendan might improve RBF and GFR in AKI even in the absence of impaired cardiac function. However, to the best of our knowledge, no study that has investigated levosimendan in this setting has been published.

To test the hypothesis that levosimendan improves renal function in patients with postoperative AKI who do not otherwise require inotropic support, we performed a blinded randomized placebo-controlled trial. Our aim was to test the effect of a short-term infusion of levosimendan on RBF and GFR. Our hypothesis was that infusion of levosimendan in early cardiac surgery-associated AKI increases both RBF and GFR.

## Patients and methods

### Patients

The study protocol was approved by the regional Ethics Committee in Gothenburg, Sweden, (Dnr 440-14, date 11/11/2014), and registered in ClinicalTrials.gov (identifier: NCT02531724, date 08/20/2015. Patients who were recovering after cardiac surgery in the cardiothoracic intensive care were screened for inclusion. The inclusion criteria were: (1) preoperative serum creatinine levels within the normal range (in women: 45–90 μmol/L, in men: 60–105 μmol/L). (2) postoperative acute kidney injury, defined as a postoperative increase (within 48 h) in serum creatinine of ≥ 27 mmol or an increase of > 50%, according to the KDIGO criteria, (3) written informed consent, (4) age > 18 years old, (5) postoperative care after open cardiac surgery with CPB. Exclusion criteria were: (1) ongoing treatment with inotropic medication (except for norepinephrine), (2) central venous saturation (ScvO_2_) < 60%, (3) impaired RBF due to vascular obstruction, e.g., from a dissection membrane. All patients were studied at the cardiothoracic intensive care unit at Sahlgrenska University Hospital, Gothenburg, Sweden. Written informed consent was obtained from all participants, or a next of kin, before initiation of the study protocol.

### Study protocol and randomization

The study is a single center, randomized, double blind, placebo-controlled cohort study. After inclusion, the patients were randomized 1:1 (sealed envelopes) to placebo or levosimendan. The randomization was stratified for gender, mechanical ventilation (yes/no), and preoperative left ventricle ejection fraction above or below 50%. A nurse, not otherwise involved in the treatment of the patient, randomized the patients and prepared the study-drug. To ensure blinding, considering that levosimendan has a yellow tint, the study drug was administered with opaque syringes and tubing, and the same volume and infusion rate was used in both the placebo and the levosimendan group.

During the study, measurements, as described below, were made before (baseline) and during treatment. The baseline value was the average of two measurements at 30 min interval before study drug administration, and the treatment measurements were the averages of three different time points, made at 3, 4 and 5 h after the start of the study drug infusion. Patients randomized to levosimendan (Simdax, Orion Pharma, Esbo, Finland) were given a bolus infusion (12 µg/kg) over 30 min, followed by 0.1 µg/kg/min for 5 h. Patients randomized to placebo received acetated Ringer’s solution (Ringers-acetate Baxter Viaflo®) infused in the same volumes and infusion rates as in the levosimendan group. During the study period, any ongoing diuretic therapy was unchanged. Norepinephrine was administered to keep the blood pressure in the target mean arterial pressure (MAP) between 70 and 80 mmHg.

### Measurements of systemic and renal variables

The measurements included MAP obtained from an arterial line (radial artery), central venous pressure (CVP) obtained from a central venous catheter (left subclavian vein or right internal jugular vein), heart rate, body temperature and urinary output. Blood gases from arterial and central venous catheters were analyzed for arterial (SaO_2_) and central venous (ScvO_2_) oxygen saturation and hematocrit. The norepinephrine dose and inspired oxygen levels were recorded. Renal perfusion pressure was calculated as MAP-CVP.

GFR was measured using infusion-clearance technique of chromium-ethylenediamine tetra acetic acid (^51^Cr-EDTA) or iohexol, and renal plasma flow (RPF) was measured using infusion-clearance of para-amino-hippurate (PAH), as described below. Due to halted hospital delivery of Cr-EDTA, iohexol was used to determine GFR from June 2019. After the collection of blood blanks, an intravenous priming dose of ^51^Cr-EDTA (GE Healthcare Limited, United Kingdom) or iohexol (Omnipaque® 300 mg I/mL; GE Healthcare, Sweden) and PAH (Merck and CO., INC, USA) were administered, followed by infusion at a constant rate, individualized to body surface area and postoperative serum creatinine. Serum concentrations of PAH and ^51^Cr-EDTA activity were measured by a spectrophotometer (Beckman DU 530; Life Science UV/Vis, USA) and a well counter (Wizard 3″ 1480, Automatic Gamma Counter; Perkin Elma LAS, Finland), respectively. Iohexol concentration was measured by ion mass spectrometry (Xevo TQMS, Waters, USA). RPF was measured using the infusion clearance technique for para-aminohippurate (PAH) and was calculated as the amount of infused PAH divided by the arterial PAH concentration, corrected for an assumed PAH extraction of 0.70, as shown by Redfors et al. in patients with AKI after cardiac surgery [8]. RBF was calculated as the renal plasma flow (RPF) divided by (1 – hematocrit). GFR was calculated as amount of infused ^51^Cr-EDTA or iohexol divided by arterial content of the substance, and filtration fraction (FF) was calculated as GFR/RPF. Renal vascular resistance (RVR) was calculated as (MAP–CVP)/RBF. Estimated GFR (eGFR) was calculated using the CKD-EPI Creatinine Equation [18]. All renal variables were normalized to a body surface area of 1.73 m^2^.

### Statistical analysis

Based on the study by Bragadottir et al. [16], we estimated that a levosimendan-induced change in GFR may be approximately 12 ml/min. To detect a difference in GFR of 12 ml/min between the groups at a power of 80% and a significance level of 5%, 12 patients per group are needed. To allow for drop-outs and skewed randomization, we aimed to enroll 30 patients. Data are presented as n (%), mean ± SD, or range. Group differences at baseline were tested using Mann–Whitney U tests or Fischer’s exact test for analysis of continuous and categorical data, respectively. A linear mixed model was used to test time and group interaction. Differences between baseline and treatment within groups were tested with Wilcoxon Signed Rank test. A *p* value of 0.05 was considered statistically significant. Predictive Analytics Software Statistics 18.0 (SPSS Inc., USA) was used for all statistical analyses.

## Results

**Fig. 1:**
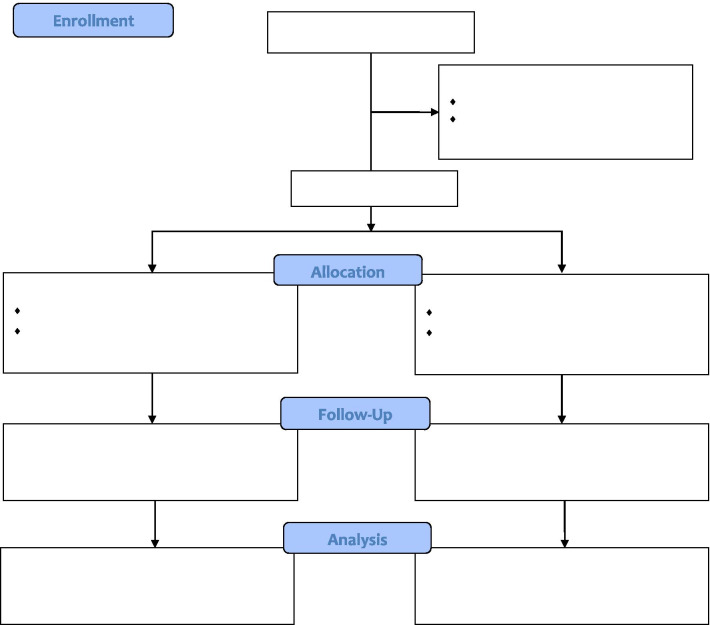
COSORT flow diagram

Between September 2015 and June 2020, 36 patients were screened. Two patients declined to participate, and four patients were excluded prior to randomization due to lack of time and bed space in the cardiothoracic ICU. One patient, randomized to placebo, did not finish the protocol due to hemodynamic instability and need for inotropic support, and was excluded from the analysis. In total, 29 patients were included in the study, see Consort flow diagram, Fig. 1.

Table 1 displays clinical and demographic characteristics of the two groups. The majority of the study participants were men and the mean preoperative creatinine was 83 ± 14 µmol/L. One patient had chronic atrial fibrillation, the remaining patients had sinus rhythm. Six patients in the placebo group had diabetes mellitus compared to one patient in the levosimendan group (*p* = 0.026). Betablocker treatment was more common in the placebo group (*p* = 0.020), but all other comorbidities and medications were comparable between the groups.

**Table 1:** Patient characteristics, preoperative

Variable	Placebo (n = 13)	Levosimendan (n = 16)	Between group difference (LMM) *p* value	
Sex, male (%)	12 (92)	10 (63)	0.906	
Mean age (year)	65 (37–78)	70 (52–82)	0.253	
Mean left ventricular ejection fraction (%)	55 ± 10	55 ± 10	1.000	
Hypertension (%)	13 (45)	12 (41)	0.107	
Mean preoperative serum creatinine (µmol/L)	82 ± 15	84 ± 13	0.628	
Betablockers	12	8	0.020	
ACE-inhibitors	4	4	1.000	
Calcium channel blockers	5	4	0.688	
Statins	9	8	0.451	
Loop diuretics	5	8	0.711	
Diabetes mellitus	6	1	0.026	
Insulin	2	0	0.192	
Peroral antidiabetics	6	1	0.026	
*Patient characteristics at study start*				
Mean creatinine at time of inclusion (µmol/L)	127 ± 22	148 ± 29	0.030	
Time from CPB to inclusion (hours)	24 ± 10	34 ± 14	0.108	
Coronary artery bypass surgery (n)	3	2	0.632	
Valve surgery (n)	3	5	0.697	
Coronary artery bypass + valve (n)	2	0	0.192	
Other (aortic dissection/double valve etc.) (n)	5	9	0.462	
CPB time (min)	117 ± 35	125 ± 52	0.965	
AKI grade 1 (n)	13	11		
AKI grade 2 (n)	0	5	0.048	

ACE: angiotensin converting enzyme. CPB: cardio pulmonary bypass. AKI: acute kidney injury, grading according to the KDIGO criteria, LMM: Linear mixed model

The patients were included at 34 ± 14 and 24 ± 10 h after the end of CPB in the levosimendan and placebo group, respectively (*p* = 0.108). At inclusion, the mean serum creatinine was higher in the patients randomized to levosimendan (148 ± 29 vs 127 ± 22 µmol/L, *p* = 0.030), and the estimated GFR (eGFR) was lower (46 ± 12 vs 57 ± 11 ml/min/1.73 m^2^, *p* = 0.025). In the levosimendan group, five patients had AKI grade 2 at inclusion, all the remaining patients of both groups had AKI grade 1 (*p* = 0.048 between groups).

### Systemic variables (Table 2)

**Table 2:** Systemic variables

	Placebo (n = 13)		Levosimendan (n = 16)		Between group difference (LMM)
Variable	Pre	Post	Pre	Post	*p* value
Central venous oxygen saturation (%)	66.1 ± 6.6	64.9 ± 4.4	67.1 ± 7.4	68.0 ± 6.4	0.402
Arterial oxygen saturation (%)	95.4 ± 1.1	95.3 ± 1.2	95.6 ± 2.0	95.2 ± 1.8	0.655
Mean arterial pressure (mmHg)	79.6 ± 11.8	80.0 ± 6.6	74.8 ± 9.2	71.7 ± 4.5	0.362
Central venous pressure (mmHg)	9.3 ± 4.3	11.0 ± 3.9 *	7.3 ± 4.3	7.5 ± 4.3	0.141
Body temperature (°C)	37.3 ± 0.5	37.4 ± 0.6	37.1 ± 0.7	37.2 ± 0.7	0.454
Norepinephrine dose (µg/kg/min)	0.031 ± 0.046	0.022 ± 0.030	0.054 ± 0.059	0.098 ± 0.113 *	0.012
Heart rate (beats/min)	83.2 ± 11.7	85.0 ± 12.0	78.6 ± 9.1	83.1 ± 12.6 *	0.250
Erythrocyte volume fraction	0.32 ± 0.04	0.31 ± 0.04 *	0.30 ± 0.03	0.30 ± 0.03	0.446

^*^ = diff paired T-test pre-post *p* < 0.05, ***p < 0.001. Between group difference (LMM) = time × group interaction from the linear mixed model

**Table 3:** Renal variables

	Placebo (n = 13)		Levosimendan (n = 16)		Between group difference (LMM)
Variable	Pre	Post	Pre	Post	*p* value
Renal blood flow (L/min)	441 ± 159	455 ± 166	327 ± 148	375 ± 156 ***	0.011
Glomerular filtration rate (ml/min)	63.7 ± 19.7	63.8 ± 18.9	47.9 ± 15.2	50.0 ± 16.1	0.440
Renal prefusion pressure (mmHg)	70.3 ± 12.9	69.1 ± 7.3	66.0 ± 7.1	64.0 ± 5.5	0.907
Renal vascular resistance (mmHg/mL/min)	0.178 ± 0.067	0.170 ± 0.061	0.253 ± 0.131	0.208 ± 0.104**	0.043
Diuresis (ml/min)	1.89 ± 1.07	1.83 ± 0.61	1.71 ± 0.87	1.82 ± 0.96	0.637
Filtration fraction	0.104 ± 0.026	0.101 ± 0.018	0.117 ± 0.044	0.104 ± 0.034**	0.056

^*^ = diff paired T-test pre-post *p* < 0.05, **p < 0.01, ***p < 0.001. Between group difference (LMM) = time × group interaction from the linear mixed model

Central hemodynamic parameters at baseline and during treatment are shown in Table 2. There were no group differences in the systemic variables at baseline. In the placebo group, CVP was increased (+ 18%, *p* = 0.009), body temperature was slightly increased (*p* = 0.036) and erythrocyte volume fraction (EVF) was slightly reduced (*p* = 0.003) during treatment. In the levosimendan group, the treatment was accompanied by increased norepinephrine dose (+ 82%, *p* = 0.007) and heart rate (+ 5.7%, *p* = 0.012), while the other systemic variables remained unchanged. No new-onset atrial fibrillation or other arrhythmias were seen during the experimental procedure.

When comparing the two groups, the norepinephrine dose was significantly higher in the levosimendan group compared to the placebo group (*p* = 0.012). Otherwise, there were no significant differences between the two groups with respect to the evolution of systemic variables.

### Renal variables (Table 3 and Fig. 2)

**Fig. 2:**
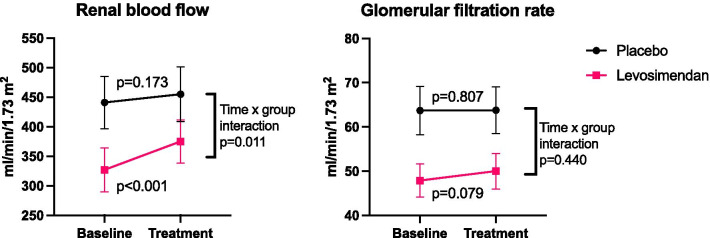
Renal blood flow and glomerular filtration rate before (baseline) and after study drug administration (treatment). *p* Values indicate difference between groups (time × group interaction from linear mixed model) and difference within groups (Wilcoxon signed rank test)

Renal variables at baseline and treatment are shown in Table 3At baseline, the patients randomized to levosimendan had a significantly lower RBF and GFR than those randomized to placebo (RBF 327 ± 148 vs 441 ± 159 ml/min/1.73m^2^, *p* = 0.045, and GFR 48.9 ± 15.2 vs 63.7 ± 19.7 ml/min/1.73 m^2^, *p* = 0.022). In the placebo group, none of the renal variables changed during the experimental procedure. In the levosimendan group, RBF was increased (+ 15%, *p* < 0.001), and renal vascular resistance (RVR) was reduced (− 18%, *p* = 0.001) during treatment. Filtration fraction was reduced by 12% (*p* = 0.003), and there was a trend towards increased GFR (+ 4.5%, *p* = 0.079) after levosimendan treatment.

Compared to the placebo group, levosimendan increased RBF (*p* = 0.011) and decreased RVR (*p* = 0.043). No group differences in GFR or other renal variables were found. In both groups, the mean serum creatinine was lower on the day after the study compared to on the inclusion, with no significant difference between the groups (*p* = 0.507). In patients with AKI grade 2 (n = 5, all in the levosimendan group), there was a mean increase in serum creatinine on the first day after the study compared to inclusion (+ 10%, n.s.).

## Discussion

In this blinded, randomized placebo-controlled trial, we studied the renal effects of the inodilator levosimendan in patients with early cardiac surgery-associated AKI, without the need for inotropic support. The main finding was that levosimendan, compared to placebo, increased RBF, due to a fall in renal vascular resistance, but had no significant impact on GFR, while filtration fraction decreased. These results suggest that in AKI, levosimendan exerts a dilation of both pre- and post glomerular resistance vessels.

To our knowledge, this is the first report on the specific renal effects of levosimendan in patients with AKI. The renal effects of levosimendan have previously studied in patients with normal renal function after cardiac surgery [16], in patients with acute heart failure [19], and in patients with the cardiorenal syndrome [17]. The data from the present study are at variance with the results of those studies. In the study by Bragadottir et al.[16], levosimendan caused a renal vasodilatation, increasing both RBF and GFR, suggesting a dilatation of predominantly the preglomerular (afferent) resistance vessels with no change in filtration fraction. Fedele et al.[19] studied the renal effects of levosimendan on RBF (assessed by renal artery Doppler) and estimated GFR (for 72 h) in patients with acute decompensated heart failure and impaired renal function. They found that levosimendan increased cardiac output, RBF and GFR, indicating that levosimendan dilated predominantly the afferent arterioles. In the study by Lannemyr et al.[17], the renal effects of levosimendan versus those of dobutamine were compared in patients with chronic heart failure and renal impairment. It was found that while both agents provided a similar increase in cardiac output and RBF, levosimendan, in contrast to dobutamine, also increased GFR. In the studies by Bragadottir [16] and Lannemyr [17], levosimendan at a dose of 0.1 µg/kg/min, increased GFR by approximately 20%. In the present study, there was a trend (*p* = 0.079) towards increased GFR with levosimendan, but the increase was modest and thus of uncertain clinical significance, and there was no significant change in diuresis.

There may be several reasons for this disparity regarding levosimendan-induced changes in GFR after cardiac surgery, as seen in the present study compared to the study of Bragadottir et al. [16]. Firstly, when compared to the placebo group, the levosimendan group in the present study had a significantly worse renal function before start of the drug infusion, despite randomization. At baseline, GFR was 25% lower in the levosimendan group with five patients in AKI class 2. One might speculate that these patients may have suffered an ongoing deterioration in GFR during the experimental procedure, which might have countered a possible levosimendan-induced improvement in GFR. Secondly, one could also speculate that the administered dose of levosimendan was not high enough to override an intense renal and preglomerular vasoconstriction, a state which has been described in patients with cardiac surgery-induced AKI [8] and as also demonstrated in the present study. Although the baseline RVR was high in the present study, particularly in the levosimendan group, levosimendan could induce a 18% decrease in RVR, an effect comparable to that seen by Bragadottir et al. [16], who showed that the same dose of levosimendan decreased RVR by 14%. Thirdly, one of the mechanisms involved in the increased RVR seen in AKI after cardiac surgery [8], is an increased activity in the renin-angiotensin system [20, 21]. Elevated levels of angiotensin II causes, in addition to its vascular effects, a contraction of glomerular mesangial cells, which induces a reduction of the glomerular ultrafiltration coefficient and thus GFR. Experimental studies have shown that levosimendan may counteract this angiotensin II-induced contraction of mesangial cells and may thus increase glomerular ultrafiltration coefficient and GFR [22]. Thus, it is possible that the previously published beneficial effects of levosimendan on GFR in patients may be mediated by inhibition of angiotensin II-induced contraction of mesangial cells, but the administered dose may have been too low to override these glomerular changes in the present study.

Renal oxygenation is determined by the balance of renal oxygen consumption and delivery. The oxygen consumption increases with increased tubular sodium load, which is closely linked to GFR [8, 23]. Since RBF, and thus renal oxygen delivery, increased to a greater extent than GFR in the present study, also reflected by a fall in renal filtration fraction, it is reasonable to assume that the renal oxygenation was improved. It has previously been demonstrated that there is a close negative correlation between renal filtration fraction and renal oxygenation [5–7].

Two major outcome studies, the LICORN [10] and LEVO-CTS [11] studies, have shown that levosimendan does not prevent major adverse outcomes after cardiac surgery, including AKI and the need for renal replacement therapy. Our findings of a lack of effect of levosimendan on GFR in postoperative AKI, are in line with the findings of those trials. In a sub-study of the CHEETAH trial [15], however, levosimendan was found to reduce the risk of AKI in a high-risk population, when used for treatment of the low cardiac output syndrome, postoperatively. Thus, one can speculate that levosimendan may exert beneficial effects on renal function only when used for treatment of heart failure as shown by the CHEETAH sub-study [15] and by our group demonstrating that levosimendan, but not dobutamine, increased GFR in patients with the cardiorenal syndrome [17].

This study has some limitations. Most importantly, it is a small study, and despite randomization, the patients in the levosimendan group had more severe AKI with lower GFR and RBF than the placebo group at inclusion, which complicates the interpretation of our results (see above). Secondly, since renal vein catheterization was not used, no data on renal oxygen consumption, renal oxygenation or individual data on renal extraction of PAH are available. The strengths of the study are that it was blinded, randomized and placebo-controlled, evaluating the renal effects of levosimendan in patients with AKI, particularly with the view that pharmacodynamic studies on renal function in AKI are almost lacking.

## Conclusions

In patients with AKI after cardiac surgery, levosimendan improved renal blood flow through renal vasodilation, but had little or no effect on GFR. The levosimendan-induced fall in renal filtration fraction suggests that levosimendan may have improved renal oxygenation. Further studies with emphasis on outcome are needed before levosimendan can be recommended for the treatment of acute kidney injury.

## Data Availability

Study data are available from the corresponding author on reasonable request.Study data are available from the corresponding author on reasonable request.

## Abbreviations

AKI: Acute kidney injury
CPB: Cardiopulmonary bypass
^51^Cr-EDTA: Chromium-ethylenediamine tetra acetic acid
CVP: Central venous pressure
GFR: Glomerular filtration rate
eGFR: Estimated glomerular filtration rate
EVF: Erythrocyte volume fraction
FF: Filtration fraction
KDIGO: Kidney Disease: Improving Global Outcomes
MAP: Mean arterial pressure
PAH: Para-amino-hippurate
RBF: Renal blood flow
RPF: Renal plasma flow
RVR: Renal vascular resistance
SaO_2_: Arterial oxygen saturation
SvO_2_: Central venous oxygen saturation
